# Determinants of Primary Nonadherence to Medications Prescribed by General Practitioners Among Adults in Hungary: Cross-Sectional Evaluation of Health Insurance Data

**DOI:** 10.3389/fphar.2019.01280

**Published:** 2019-10-31

**Authors:** Nouh Harsha, László Kőrösi, Anita Pálinkás, Klára Bíró, Klára Boruzs, Róza Ádány, János Sándor, Árpád Czifra

**Affiliations:** ^1^Department of Preventive Medicine, Faculty of Public Health, University of Debrecen, Debrecen, Hungary; ^2^Department of Financing, National Health Insurance Fund, Budapest, Hungary; ^3^Department of Health Systems Management and Quality Management in Health Care, Faculty of Public Health, University of Debrecen, Debrecen, Hungary

**Keywords:** medication adherence, dispensed prescriptions, urbanization, level of education, GP vacancy, GMP size, geographical inequality, exemption certificate

## Abstract

**Background:** Primary nonadherence to prescribed medications occurs when patients do not fill or dispense prescriptions written by healthcare providers. Although it has become an important public health issue in recent years, little is known about its frequency, causes, and consequences. Moreover, the pattern of risk factors shows remarkable variability across countries according to the published results. Our study aimed to assess primary nonadherence to medications prescribed by general practitioners (GPs) and its associated factors among adults in Hungary for the period of 2012–2015.

**Methods:** Data on all general medical practices (GMPs) of the country were obtained from the National Health Insurance Fund and the Central Statistical Office. The ratio of the number of dispensed medications to the number of prescriptions written by a GP for adults was used to determine the medication adherence, which was aggregated for GMPs. The effect of GMP characteristics (list size, GP vacancy, patients’ education provided by a GMP, settlement type [urban or rural], and geographical location [by county] of the center) on adherence, standardized for patients’ age, sex, and eligibility for an exemption certificate, were investigated through generalized linear regression modeling.

**Results:** A total of 281,315,386 prescriptions were dispensed out of 438,614,000 written by a GP. Overall, 64.1% of prescriptions were filled. According to the generalized linear regression coefficients, there was a negative association between standardized adherence and urban settlement type (*b* = -0.099, 95%CI = -0.103 to -0.094), higher level of education (*b* = -0.440, 95%CI = -0.468 to -0.413), and vacancy of the general practices (*b* = -0.193, 95%CI = -0.204 to -0.182). The larger GMP size proved to be a risk factor, and there was a significant geographical inequality for counties as well.

**Conclusions:** More than one-third of the written prescriptions of GPs for adults in Hungary were not dispensed. This high level of nonadherence had great variability across GMPs, and can be explained by structural characteristics of GMPs, the socioeconomic status of patients provided, and the quality of cooperation between patients and GPs. Moreover, our findings suggest that the use of the dispensed-to-prescribed medication ratio in routine monitoring of primary health care could effectively support the necessary interventions.

## Introduction

Recently, the concept of adherence to medication has gained increasing attention (Ho et al., 2009; Lee et al., 2018) and has become an important public health issue (Balkrishnan, 2005; Fischer et al., 2010). The WHO recommends the use of the dispensed proportion of prescribed drugs, the primary adherence to medication, as a basic indicator of health care operation (World Health Organization, 1993). Indeed, medication adherence is considered the cornerstone in the management, control, and prevention of loss of the desired therapeutic outcome, disease progression, and complications (De Simoni et al., 2013; Colombo et al., 2016; Nielsen et al., 2017). Several studies attributed higher morbidity and mortality among patients to medication nonadherence (Ho et al., 2006a; Ho et al., 2006b; Aronson, 2007; Gwadry-Sridhar et al., 2013). In addition, nonadherence increases healthcare cost and hospitalization and is associated with deterioration of the quality of life (Osterberg and Blaschke, 2005; Sokol et al., 2005; Brown and Bussell, 2011; Raebel et al., 2012; Kardas, 2013).

Nonadherence is a global issue (Cramer, 2004; Stankuniene et al., 2012; Kleinsinger, 2018). A meta-analysis of 20 studies conducted between 1998 and 2010 in Australia, Canada, the USA, and Europe to assess the extent of adherence of cardiovascular patients to their regimens indicated that approximately 50% were not properly adherent to cardiovascular medications prescribed for preventive purposes (Naderi et al., 2012). However, a meta-analysis of hundreds of studies conducted over a 50-year period reported that, on average, approximately one-fourth of the patients did not adhere to their regimens (DiMatteo, 2004). Furthermore, a study conducted in Canada in primary healthcare between 2006 and 2009 reported that 31.3% of the written prescriptions were not filled (Tamblyn et al., 2014). In 2018, an extensive meta-analysis conducted to measure primary nonadherence to medications of chronic diseases reported a nonadherence rate of 14.6% (Lemstra et al., 2018).

Several factors have been identified to affect patient adherence to prescribed regimens (Lee et al., 2017; Lee et al., 2018). Those factors include patients’ demographic (age and sex) and socioeconomic status (e.g., education, income, and social support) (Krousel-Wood et al., 2004; Park et al., 2008; Peek et al., 2016; Boruzs et al., 2016; Nashilongo et al., 2017). In addition, patients’ cognitive ability, expectations, beliefs, attitude, comorbidities, and poly-pharmacy were reported to be major determinants (Vermeire et al., 2001; Mardby et al., 2007; Ferdinand et al., 2017; Rampamba et al., 2018). Furthermore, factors linked to the healthcare system (Kardas et al., 2013; Granger et al., 2015) and treatment-related issues (side effects and therapy duration) were found to affect levels of adherence (Fried et al., 2011).

Some interventions tested globally to enhance adherence included simple dose adjustment and reduction of the number of medications (Burke et al., 1997; Schedlbauer et al., 2010), reminders and improved scheduling (Claxton et al., 2001), and educational interventions (Kuntz et al., 2014; Torres-Robles et al., 2018). Other interventions included more comprehensive and complex strategies, such as expansion of the pharmacist role in health care, enhancement of patient–health care provider communication and trust, provision of services (Lu et al., 2008; Banning, 2009), proper description of disease and medications (Osterberg and Blaschke, 2005; Huang et al., 2017), habit analysis and management of side effects (Omran et al., 2012; Conn and Ruppar, 2017), patient follow-up, social and behavioral support and motivation (DiMatteo et al., 2012; Varshney, 2013; Kuntz et al., 2014; Ferdinand et al., 2017), and acting on patients’ feedback (Farris et al., 2016).

Although adherence to medications is crucial for successful treatment strategies, less attention has been given to this issue until recently (Storm et al., 2008; Joyce, 2010). The lack of detailed knowledge on the frequency, causes, and consequences of nonadherence obstructs the elaboration of interventions (Fischer et al., 2010; Shin et al., 2012).

Nonadherence in Hungary has so far been little investigated. According to the self-reported data of the European Social Survey (Larsen et al., 2009), 18% of participants did not adhere precisely to the prescribed regimens in Europe. The nonadherence rate detected in this survey was 20.3% in Hungary. This fourth highest rate among the 24 surveyed countries (with reported rates ranging from 6.3% to 25.2%) suggests that nonadherence is of especially high importance in Hungary (Stavropoulou, 2011). It seems plausible that this relatively high nonadherence rate contributes to the relatively high amenable mortality in Hungary, which is 2.1 times higher than the average in the European Union, according to the EUROSTAT statistics for 2015 (). Since the Hungarian primary health care performance is ranked in the weakest third in Europe (Kringos et al., 2013), general medical practices may be a potential target of intervention to improve adherence and thereby to improve the public health status in the country. It is worth mentioning that the health insurance in Hungary, provided by the National Health Insurance Fund (NHIF), covers more than 96% of the population. The costs of medicines are shared by the NHIF and the patient by a health insurance scheme. Due to this cost sharing, patients are not motivated to hide any drug consumption from the NHIF (Gaál et al., 2011). Thus, its data on prescribed and dispensed drugs are reliable and can be used to assess adherence to medications in a nationally representative manner.

The aims of this study were 1) to describe adherence to general practitioner (GP)-prescribed medications among Hungarian adults by quantifying the dispensed proportion of prescribed medications, 2) to describe the variability of this adherence across general medical practices, and 3) to assess the influence of general medical practices’ structure and patients’ characteristics on that variability. For this study, we hypothesized that nonadherence to GP-prescribed medications among adults in Hungary is related to patients’ characteristics (age, sex, eligibility for exemption certificate) and general medical practice characteristics such as socioeconomic status of adults provided (indicated by standardized relative education), list size of general medical practice, GP vacancy, type of settlement, and location by county where general medical practice is located.

## Methods

### Setting

This study was a secondary database analysis. The unit of analysis was the prescription written by a GP and dispensed by patients. Data on written and dispensed prescriptions were obtained from the NHIF. All general medical practices (GMPs) operating in Hungary were investigated. The available data covered the period from the beginning of 2012 until the end of the third quarter of 2015.

### Data

These data were stratified by age (5-year bands), sex, and possession of exemption certificate. Drugs were classified according to the Anatomical Therapeutic Chemical (ATC) Classification recommended by the WHO (WHO Collaborating Center for Drug Statistics Methodology). ATC groups of “Antineoplastic and immunomodulating agents” and “Antiparasitic products, insecticides, and repellents” were not included in the analyses because the use of these drugs is not connected to GPs according to the Hungarian regulations.

The NHIF also provided data on the characteristics of GMPs. GMPs were described by the number of adults for which care was provided using the official categorization of the NHIF (less than 800, 801–1,200, 1,201–1,600, 1,601–2,000, and 2,001 or more patients). GMPs were also categorized as having a vacant or filled GP position (provided by a temporary GP with availability restricted in time and place or by a permanent contracted GP with continuous availability). Rural or urban GMPs were distinguished. GMPs were classified according to their geographical location by county.

The socioeconomic status of the adults receiving care at each GMP was approximated by the sex- and age-internally standardized relative education. This indicator was computed by indirect method, using sex and age group-specific levels of education from the 2011 Hungarian Census and the sex and age group composition of GMP clients (Janka et al., 2018). Since internal standardization was applied, the national average of socioeconomic status corresponds to value 1 of standardized relative education.

### Statistical Analysis

The sex-, age-, and exemption certificate-specific number of written and dispensed prescriptions and the percentage of drugs actually dispensed (dispensed to written ratio, DWR) as the indicator for medication adherence (World Health Organization, 1993) were determined by the NHIF for the whole country for the investigated period. These national reference DWRs were calculated for each studied ATC group. NHIF data on prescribed and dispensed drugs are reliable and the statistical power could be ensured by the applied design being strong.

Our dependent variables were adherence ratios indirectly standardized for age, sex, and exemption certificate. These standardized adherence ratios were calculated by dividing the agregated GMP-specific numbers of the observed dispensed prescriptions by the aggregated GMP-specific numbers of expected dispensed prescriptions.

Using the sex-, age-, and exemption certificate-specific number of written prescriptions by GMP and the national reference DWRs, the expected number of dispensed prescriptions was calculated for each GMP (summarizing the expected number of dispensed prescriptions across strata). The ratio of the registered number of filled prescriptions in a GMP and the calculated GMP-specific expected number of dispensed prescriptions was computed as the GMP-specific standardized DWR (SDWR) for each ATC group.

The distributions of the ATC group-specific and the general SDWR values computed for GMP were not normal according to Kolmogorov–Smirnov testing (**Supplementary Appendix 14**). Median values and interquartile ranges were used to describe their distribution. Taking into consideration that calculated SDWRs were positive numbers, Box–Cox transformation was applied to normalize the SDWRs (Curran-Everett, 2018) (histograms for original and transformed SDWR parameters are in **Supplementary Appendix 15**).

To identify the factors that influence the SDWR by controlling for the time, we conducted generalized linear modeling. Linear regression coefficients (*b*) with the corresponding 95% confidence interval (95%CI) were calculated. In the model, GP vacancy and settlement type were used as binary parameters, and county location and GMP size were used as dummy variables with reference categories of Budapest and GMP size of 1,601–2,000 persons, respectively. This analysis was also conducted for each ATC group. Pearson’s chi-square goodness-of-fit test was applied to describe the performance of regression modeling.

The level of significance was fixed at 95% (*p* < 0.05). SPSS version 20 was used in the data analysis.

## Results

The distribution of characteristics for the 4,856 GMPs investigated is summarized in **Table 1**. Of the studied GMPs, 3.30% were vacant, and 34.70% were in rural areas. The majority of GMPs provided care to 1,201–1,600 (30.5%) or 1,601–2,000 (31.7%) adults. Due to the internal approach of indirect standardization, the mean relative education of the provided population was 1.00 (SD ± 0.10).

**Table 1:** Distribution of general medical practice (GMP) characteristics in Hungary.

Variable name		Number of GMP (%)
Vacancy of general practitioner	Vacant	160 (3.3)
	Fulfilled	4,696 (96.7)
Type of settlement	Rural	1,683 (34.7)
	Urban	3,173 (65.3)
Size of GMP	<800	158 (3.3)
	800–1,200	677 (13.9)
	1,201–1,600	1,481 (30.5)
	1,601–2,000	1,541 (31.7)
	> 2,000	999 (20.6)
County	Budapest	875 (18)
	Baranya	209 (4.3)
	Bács-Kiskun	256 (5.3)
	Békés	191 (3.9)
	Borsod-Abaúj-Zemplén	377 (7.8)
	Csongrád	205 (4.2)
	Fejér	196 (4)
	Gyo˝r	205 (4.2)
	Hajdú-Bihar	246 (5.1)
	Heves	161 (3.3)
	Komárom-Esztergom	146 (3)
	Nógrád	109 (2.2)
	Pest	477 (9.8)
	Somogy	177 (3.6)
	Szabolcs-Szatmár-Bereg	267 (5.5)
	Jász-Nagykun-Szolnok	194 (4)
	Tolna	121 (2.5)
	Vas	134 (2.8)
	Veszprém	169 (3.5)
	Zala	141 (2.9)
Total number of GMPs		4,856 (100.0)

In general, the observed DWR was 64.1% (**Table 2**). There was only a slight difference between men (63.6%) and women (64.5%). Age showed considerable influence. The lowest DWR was registered for middle-aged adults (62.1%). The highest was detected among adults with exemption certificates (78.3%). Regarding sex and exemption certificate, the same pattern was observed for each ATC group, while the age dependency of the DWR varied by ATC group (details in **Supplementary Appendices 1–12**).

**Table 2:** Dispensed to written prescription ratio by patient characteristics in Hungary in the period from 1 January 2012 to 30 September 2015.

Patient characteristics		Written prescriptions	Dispensed prescriptions	Percentage of the dispensed prescriptions
Age groups (years)	18–44	39,971,036	25,539,871	63.9
	45–64	171,996,562	106,753,470	62.1
	65 and above	226,646,402	149,022,045	65.8
Sex	Male	172,358,931	109,603,855	63.6
	Female	266,255,069	171,711,531	64.5
Exemption certificate	Yes	47,960,440	37,548,944	78.3
	No	390,653,560	243,766,442	62.4
Total		438,614,000	281,315,386	64.1

There was wide variability of the DWR by drug class (**Table 3**). The lowest DWR was registered for cardiovascular system drugs at 59.4%. The highest value was 79.1%, reported for the anti-infective drugs for systemic use.

**Table 3:** Dispensed to written prescription ratio by ATC group in Hungary in the period from 1 January 2012 to 30 September 2015.

	Written prescriptions	Dispensed prescriptions	Percentage of the dispensed prescriptions
Alimentary tract and metabolism	71,736,340	49,208,724	68.6
Blood and blood forming organs	30,237,857	20,921,060	69.2
Cardiovascular system	228,225,210	135,489,086	59.4
Dermatologicals	4,382,185	2,707,111	61.8
Genitourinary system and sex hormones	3,396,924	2,289,588	67.4
Systemic hormonal preparations*	4,822,082	3,570,757	74.1
Antiinfectives for systemic use	13,348,380	10,559,995	79.1
Musculoskeletal system	29,820,787	20,541,099	68.9
Nervous system	28,337,910	20,040,657	70.7
Respiratory system	21,830,141	14,357,120	65.8
Sensory organs	1,626,640	1,113,623	68.5
Various	849,544	516,566	60.8
Altogether^	438,614,000	281,315,386	64.1

*Excluding sex hormones and insulin.

^ATC groups of “Antineoplastic and immunomodulating agents” and “Antiparasitic products, insecticides, and repellents” were not studied.

The SDWRs varied over a wide range. The limits of the interquartile range were 0.87 and 1.22. The interquartile ranges for the ATC groups varied between 0.14 (anti-infectives for systemic use) and 0.42 (cardiovascular system agents) apart from the various (ATC V) groups (**Supplementary Appendix 13**).

An inverse association was found between normalized SDWRs (*D* = 0.002; *p* = 0.200 according to the Kolmogorov–Smirnov test as presented in **Supplementary Appendix 14**) and the relative education of the adult population that received care (*b* = -0.440, 95%CI = -0.468 to -0.413), vacancy of GP (*b* = -0.193, 95%CI = -0.204 to -0.182), and urban residential place (*b* = -0.099, 95%CI = -0.103 to -0.094). The size of the GMP proved to be a significant influencing factor. A relatively higher SDWR was observed in smaller GMPs (*b* *_X_*_–800_ = 0.052, 95%CI = 0.041–0.063, *b*_801–1200_ = 0.031, 95%CI = 0.025–0.037, *b*_1201–1600_ = 0.017, 95%CI = 0.013–0.022), while the larger GMPs showed a lower SDWR (*b*_2001–_ *_X_* = -0.014, 95%CI = -0.019 to -0.009).

There was a significant impact of geographical location according to the county-specific regression coefficients (**Table 4**). According to the generalized linear regression coefficients, the urban residential place, high relative education, and vacant GP position (in decreasing effect size) are the stronger factors that reduce the GMP-level SDWR. This general pattern was observed for each studied ATC group (**Supplementary Appendix 16**).

**Table 4:** Generalized linear modeling^ on the associations between general medical practice (GMP) characteristics and normalized* age-, gender-, and exemption certificate-standardized dispensed to written prescription ratios among Hungarian adults in the period from 1 January 2012 to 30 September 2015.

	Regression coefficient [95%CI]
Standardized relative education**	–0.440 [-0.468; -0.413]
Vacant GP position/filled GP position	–0.193 [-0.204; -0.182]
Urban/rural	–0.099 [-0.103; -0.094]
*X*–800 GMP size/1,601–2,000 GMP size	0.052 [0.041; 0.063]
801–1,200 GMP size/1,601–2,000 GMP size	0.031 [0.025; 0.037]
1,201–1,600 GMP size/1,601–2,000 GMP size	0.017 [0.013; 0.022]
2,001–*X* GMP size/1,601–2,000 GMP size	–0.014 [-0.019; -0.009]
Baranya county/Budapest	0.083 [0.072; 0.093]
Bács-Kiskun county/Budapest	0.056 [0.046; 0.067]
Békés county/Budapest	0.023 [0.012; 0.033]
Borsod-Abaúj-Zemplén county/Budapest	–0.015 [-0.024; -0.006]
Csongrád county/Budapest	0.021 [0.011; 0.032]
Fejér county/Budapest	–0.132 [-0.142; -0.121]
Gyo˝r-Moson-Sopron county/Budapest	–0.002 [-0.012; 0.008]
Hajdú-Bihar county/Budapest	–0.035 [-0.045; -0.025]
Heves county/Budapest	–0.033 [-0.044; -0.021]
Jász-Nagykun-Szolnok county/Budapest	0.044 [0.033; 0.055]
Komárom-Esztergom county/Budapest	0.072 [0.060; 0.084]
Nógrád county/Budapest	–0.028 [-0.042; -0.015]
Pest county/Budapest	–0.002 [-0.01; 0.006]
Somogy county/Budapest	0.079 [0.068; 0.09]
Szabolcs-Szatmár-Bereg county/Budapest	0.074 [0.064; 0.084]
Tolna county/Budapest	0.060 [0.047; 0.073]
Vas county/Budapest	0.103 [0.091; 0.115]
Veszprém county/Budapest	0.006 [-0.006; 0.017]
Zala county/Budapest	0.017 [0.005; 0.029]

*Box–Cox transformation.

**Standardized continuous parameter.

^p = 0.060 (Pearson’s chi-square goodness-of-fit test).

## Discussion

Our investigation, which is the first population-based study in Hungary on medication nonadherence, found that only 64.1% of the medicines prescribed by a GP in Hungary are dispensed. This kind of adherence varies from 59.4% (cardiovascular systems’ agents) to 79.1% (anti-infectives for systemic use) by ATC groups. This registered nonadherence (35.9%) is higher than the levels of nonadherence observed in Canada (31.3%), Woonsocket, USA (24%), Massachusetts, USA (22.5%), Scotland (14.5%), and Sweden (2.4%) (Beardon et al., 1993; Ekedahl and Mansson, 2004; Fischer et al., 2010; Fischer et al., 2011; Tamblyn et al., 2014).

Gender has proven to be not an important determinant of nonadherence, while middle-aged adults are at risk of not dispensing prescribed medications. The strong protective role of eligibility for exemption certificates demonstrates the influence of patients’ socioeconomic status.

According to our regression models, the urban environment is a risk factor for nonadherence. There are publications with similar (Kim et al., 2016) and opposite (Park et al., 2008; Karakurt and Kasikci, 2012) conclusions. Certain elements of the urban lifestyle (better access to health services, better economic conditions, special working schedule, and urban attitude and beliefs) supposed to underlie these variable observations (Nair et al., 2011; Lewis et al., 2012; Khan et al., 2014; Magnabosco et al., 2015; Boruzs et al., 2018) urge further investigations in Hungary as key obstacles to medication adherence.

Our study found a relatively strong negative association between education and the level of adherence. This finding is consistent with findings reported by the second round of the European Social Survey (Larsen et al., 2009), but there are publications on the positive association between education and medication adherence, as well (Karakurt and Kasikci, 2012; Daniel and Veiga, 2013). A higher level of education can be an indicator of a better ability to perceive the benefit of GPs’ instructions (Mardby et al., 2007). However, the effect of education on adherence is complex (Isacson and Bingefors, 2002), and not only knowledge but also the education-related attitude plays an important role (Andersen, 1995). Highly educated people hold themselves accountable for their own health (Hashimoto and Fukuhara, 2004) and are more critical towards GPs’ instructions (Gudjonsson and Sigurdsson, 2003). Our observation could reflect a poor collaboration between patients and GPs, which counterbalances the better understanding of medications’ benefit.

Our study demonstrated the adherence-decreasing role of GP vacancies, when a permanent GP replacing the missing GP provides care. A similar finding was reported in South Korea (Park et al., 2008). This effect can be straightforwardly explained by the well-known importance of the continuity of care and trust in physicians (Rost et al., 1990; Hjortdahl and Laerum, 1992; Mainous et al., 2001). On the other hand, this result demonstrates that the workforce crisis in primary health care (resulting in increasing number of GP vacancies) exerts its detrimental effect on patients’ prognosis (Sandor et al., 2018) through reduced adherence to medication.

The importance of geographical inequalities in medication adherence (Couto et al., 2014; Di Martino et al., 2016) has been confirmed by our results. The county in our context can be considered a summary indicator of many spatially variable factors not related to urbanization, level of education, GP vacancy, or socioeconomic status (controlled by standardization for sex, age, and eligibility for an exemption certificate). The importance of the availability of health services, not controlled for our investigation, may be reflected in this observation (Tan et al., 2017). According to our results, this impact is less than the impact of GP vacancy, level of education, and urban living environment.

Our study could demonstrate the importance of the GMP list size: the more patients belong to a GMP, the lower the medication adherence. Taking into consideration that one GP and one nurse represent the typical staff in a Hungarian GMP (Gaál et al., 2011), this effect may be simply attributed to the less time that can be devoted to a patient.

### Implications

The observed high medication nonadherence obviously contributes to the impaired prognosis of diseases that can be treated by medications available. Although this impact could not be quantified in this investigation, taking into consideration the 59.4% observed adherence for cardiovascular diseases (which basically determines the life expectancy in European countries), this impact can be great, requiring further and more detailed studies and likely urging interventions.

On the other hand, the work, time, and capacities behind a prescription not filled contribute to the waste of resources in health care. Therefore, the observed nonadherence can also be considered an indicator of the effectiveness of patient–GP cooperation. The wide variability in GMP-level adherence we observed suggests that primary health care (PHC) teams have variable effectiveness in this respect. Furthermore, our results demonstrate that the role of GPs is especially weak in managing patients’ diseases among urban and higher educated adults. Consequently, the PHC monitoring system needs to be completed with primary medication adherence monitoring to identify GMPs to be improved and to benchmark the necessary interventions including, *inter alia*, educational interventions and improving patient physician relationship and communication.

## Strengths and Limitations

The health insurance in Hungary, provided by the NHIF, covers more than 96% of the population. The costs of medicines are shared by the NHIF and the patient by a health insurance scheme. Due to this cost sharing, patients are not motivated to hide any drug consumption from the NHIF (Gaál et al., 2011). Thus, its data on prescribed and dispensed drugs are reliable and can be used to assess adherence to medications in a nationally representative manner. The statistical power could be ensured by the applied design being strong.

Although drug dispensing estimation is an objective process and is considered the most reliable method for assessing adherence in integrated healthcare systems, this approach cannot address other aspects of adherence (drug application according to the prescribed regime). In fact, our study gives an idea of patient’s initial decision to remain on prescribed medications. Furthermore, many important factors that can influence adherence, such as side effects, comorbidity and polypharmacy, access to healthcare, and patient attitudes, were not available through the analyzed database and were not included in our study model, which restricts the convincing power of the observed associations, obviously. Further research with more explanatory parameters is required to improve the relevance of our findings. Finally, our observations need confirmation by studies with individual-level data on patient characteristics.

## Conclusions

More than one-third of the written prescriptions of GPs for adults were not dispensed in Hungary. This high level of nonadherence was realized with a wide variability across GMPs can be explained by structural characteristics of GMPs, the socioeconomic status of patients provided, and the quality of cooperation between patients and GPs. Moreover, our findings suggest that the use of the dispensed-to-prescribed medication ratio in routine monitoring of primary health care can effectively support the necessary interventions. Since we have determined an alarmingly high level of nonadherence across the country, and given that nonadherence has a multifactorial etiology, our study urges further investigations to determine the main obstacles to primary nonadherence.

## Data Availability Statement

The datasets used and/or analyzed during the current study are available from the corresponding author on reasonable request.

## Ethics Statement

According to the Hungarian legislation, there was no need for ethical approval to implement the secondary analysis of the study.

## Author Contributions

NH: literature review, data analysis, interpretation of results, writing paper. LK and AP: prepared the primary database. RÁ, KBí, and KBo: conception and interpretation of results. JS: elaboration of design, conception, interpretation of results, finalizing manuscript. ÁC: interpretation of results, finalizing manuscript.

## Funding

The reported study was carried out in the framework of the “Public Health Focused Model Programme for Organising Primary Care Services Backed by a Virtual Care Service Centre” (SH/8/1). The Model Programme is funded by the Swiss Government *via* the Swiss Contribution Programme (SH/8/1) in agreement with the Government of Hungary. Additional source of funding was from GINOP-2.3.2-15-2016-00005 project which was co-financed by the European Union and the European Regional Development Fund, and by the Stipendium Hungaricum Scholarship Programme (grant 124219 to NH).

## Conflict of Interest

The authors declare that the research was conducted in the absence of any commercial or financial relationships that could be construed as a potential conflict of interest.

## Abbreviations

ATC, Anatomical Therapeutic Chemical; *b*, unstandardized regression coefficient; CI, Confidence interval; DWR, dispensed to written ratio; GMP, General medical practice; GP, General practitioner; NHIF, National Health Insurance Fund; SDWR, standardized dispensed to written ratio; WHO, World Health Organization.
