# Supplementary material for: Determinants of Primary Nonadherence to Medications Prescribed by General Practitioners Among Adults in Hungary: Cross-Sectional Evaluation of Health Insurance Data
Source: Front Pharmacol. 2019 Oct 31;10:1280. doi: 10.3389/fphar.2019.01280 (PMC6836763; doi:10.3389/fphar.2019.01280)
Supplement: Supplementary file 1 [file DataSheet_1.docx]

# Supplementary Material

**Appendix 1.** Dispensed to written prescription ratio for ATC A drugs (alimentary tract and metabolism agents) by patient characteristics in Hungary in the period from 1 January 2012 to 30 September 2015

| Patient characteristics | | Written prescriptions | Dispensed prescriptions | Percentage of the dispensed prescriptions |
| --- | --- | --- | --- | --- |
| Age groups (years) | 18-44 | 6174881 | 4104388 | 66.5% |
|  | 45-64 | 27880143 | 18857038 | 67.6% |
|  | 65 and above | 37681316 | 26247298 | 69.7% |
| Sex | Male | 26771874 | 18416334 | 68.8% |
|  | Female | 44964466 | 30792390 | 68.5% |
| Exemption certificate | Yes | 9362841 | 7377434 | 78.8% |
|  | No | 62373499 | 41831290 | 67.1% |
| Total | | 71736340 | 49208724 | 68.6% |

**Appendix 2.** Dispensed to written prescription ratio for ATC B drugs (blood and blood forming organs agents) by patient characteristics in Hungary in the period from 1 January 2012 to 30 September 2015

| Patient characteristics | | Written prescriptions | Dispensed prescriptions | Percentage of the dispensed prescriptions |
| --- | --- | --- | --- | --- |
| Age groups (years) | 18-44 | 1707816 | 1084488 | 63.5% |
|  | 45-64 | 10733646 | 7219083 | 67.3% |
|  | 65 and above | 17796395 | 12617489 | 70.9% |
| Sex | Male | 13094524 | 9091791 | 69.4% |
|  | Female | 17143333 | 11829269 | 69% |
| Exemption certificate | Yes | 3357110 | 2682641 | 79.9% |
|  | No | 26880747 | 18238419 | 67.8% |
| Total | | 30237857 | 20921060 | 69.2% |

**Appendix 3.** Dispensed to written prescription ratio for ATC C drugs (cardiovascular system agents) by patient characteristics in Hungary in the period from 1 January 2012 to 30 September 2015

| Patient characteristics | | Written prescriptions | Dispensed prescriptions | Percentage of the dispensed prescriptions |
| --- | --- | --- | --- | --- |
| Age groups (years) | 18-44 | 12904985 | 7072587 | 54.8% |
|  | 45-64 | 91945735 | 52144529 | 56.7% |
|  | 65 and above | 123374490 | 76271970 | 61.8% |
| Sex | Male | 92185977 | 54097172 | 58.7% |
|  | Female | 136039233 | 81391914 | 59.8% |
| Exemption certificate | Yes | 18623266 | 14488200 | 77.8% |
|  | No | 209601944 | 121000886 | 57.7% |
| Total | | 228225210 | 135489086 | 59.4% |

**Appendix 4.** Dispensed to written prescription ratio for ATC D drugs (dermatological agents) by patient characteristics in Hungary in the period from 1 January 2012 to 30 September 2015

| Patient characteristics | | Written prescriptions | Dispensed prescriptions | Percentage of the dispensed prescriptions |
| --- | --- | --- | --- | --- |
| Age groups (years) | 18-44 | 1007238 | 639052 | 63.4% |
|  | 45-64 | 1736860 | 1074165 | 61.8% |
|  | 65 and above | 1638087 | 993894 | 60.7% |
| Sex | Male | 1717976 | 1054609 | 61.4% |
|  | Female | 2664209 | 1652502 | 62% |
| Exemption certificate | Yes | 856917 | 590367 | 68.9% |
|  | No | 3525268 | 2116744 | 60% |
| Total | | 4382185 | 2707111 | 61.8% |

**Appendix 5.** Dispensed to written prescription ratio for ATC G drugs (genitourinary system and sex hormones agents) by patient characteristics in Hungary in the period from 1 January 2012 to 30 September 2015

| Patient characteristics | | Written prescriptions | Dispensed prescriptions | Percentage of the dispensed prescriptions |
| --- | --- | --- | --- | --- |
| Age groups (years) | 18-44 | 226801 | 164769 | 72.6% |
|  | 45-64 | 821421 | 541791 | 66% |
|  | 65 and above | 2348702 | 1583028 | 67.4% |
| Sex | Male | 2252287 | 1521759 | 67.6% |
|  | Female | 1144637 | 767829 | 67.1% |
| Exemption certificate | Yes | 434919 | 337760 | 77.7% |
|  | No | 2962005 | 1951828 | 65.9% |
| Total | | 3396924 | 2289588 | 67.4% |

**Appendix 6.** Dispensed to written prescription ratio for ATC H drugs (systemic hormonal preparations, excluding sex hormones and insulins) by patient characteristics in Hungary in the period from 1 January 2012 to 30 September 2015

| Patient characteristics | | Written prescriptions | Dispensed prescriptions | Percentage of the dispensed prescriptions |
| --- | --- | --- | --- | --- |
| Age groups (years) | 18-44 | 881132 | 660897 | 75% |
|  | 45-64 | 2088223 | 1542626 | 73.9% |
|  | 65 and above | 1852727 | 1367234 | 73.8% |
| Sex | Male | 790899 | 570827 | 72.2% |
|  | Female | 4031183 | 2999930 | 74.4% |
| Exemption certificate | Yes | 434500 | 339267 | 78.1% |
|  | No | 4387582 | 3231490 | 73.7% |
| Total | | 4822082 | 3570757 | 74.1% |

**Appendix 7.** Dispensed to written prescription ratio for ATC J drugs (anti-infectives for systemic use) by patient characteristics in Hungary in the period from 1 January 2012 to 30 September 2015

| Patient characteristics | | Written prescriptions | Dispensed prescriptions | Percentage of the dispensed prescriptions |
| --- | --- | --- | --- | --- |
| Age groups (years) | 18-44 | 6072287 | 4545158 | 74.9% |
|  | 45-64 | 4500908 | 3690082 | 82% |
|  | 65 and above | 2775185 | 2324755 | 83.8% |
| Sex | Male | 4779654 | 3708273 | 77.6% |
|  | Female | 8568726 | 6851722 | 80% |
| Exemption certificate | Yes | 1319135 | 1124071 | 85.2% |
|  | No | 12029245 | 9435924 | 78.4% |
| Total | | 13348380 | 10559995 | 79.1% |

**Appendix 8.** Dispensed to written prescription ratio for ATC M drugs (musculoskeletal system agents) by patient characteristics in Hungary in the period from 1 January 2012 to 30 September 2015

| Patient characteristics | | Written prescriptions | Dispensed prescriptions | Percentage of the dispensed prescriptions |
| --- | --- | --- | --- | --- |
| Age groups (years) | 18-44 | 3695766 | 2456246 | 66.5% |
|  | 45-64 | 12483264 | 8487634 | 68% |
|  | 65 and above | 13641757 | 9597219 | 70.4% |
| Sex | Male | 12456342 | 8517643 | 68.4% |
|  | Female | 17364445 | 12023456 | 69.2% |
| Exemption certificate | Yes | 4669932 | 3738666 | 80.1% |
|  | No | 25150855 | 16802433 | 66.8% |
| Total | | 29820787 | 20541099 | 68.9% |

**Appendix 9.** Dispensed to written prescription ratio for ATC N drugs (nervous system agents) by patient characteristics in Hungary in the period from 1 January 2012 to 30 September 2015

| Patient characteristics | | Written prescriptions | Dispensed prescriptions | Percentage of the dispensed prescriptions |
| --- | --- | --- | --- | --- |
| Age groups (years) | 18-44 | 2599090 | 1870767 | 72% |
|  | 45-64 | 9879005 | 6779307 | 68.6% |
|  | 65 and above | 15859815 | 11390583 | 71.8% |
| Sex | Male | 8716855 | 6215523 | 71.3% |
|  | Female | 19621055 | 13825134 | 70.5% |
| Exemption certificate | Yes | 4700421 | 3689171 | 78.5% |
|  | No | 23637489 | 16351486 | 69.2% |
| Total | | 28337910 | 20040657 | 70.7% |

**Appendix 10.** Dispensed to written prescription ratio for ATC R drugs (respiratory system agents) by patient characteristics in Hungary in the period from 1 January 2012 to 30 September 2015

| Patient characteristics | | Written prescriptions | Dispensed prescriptions | Percentage of the dispensed prescriptions |
| --- | --- | --- | --- | --- |
| Age groups (years) | 18-44 | 4239364 | 2641667 | 62.3% |
|  | 45-64 | 9121470 | 5890282 | 64.6% |
|  | 65 and above | 8469307 | 5825171 | 68.8% |
| Sex | Male | 8666122 | 5800762 | 66.9% |
|  | Female | 13164019 | 8556358 | 65% |
| Exemption certificate | Yes | 3710124 | 2832161 | 76.3% |
|  | No | 18120017 | 11524959 | 63.6% |
| Total | | 21830141 | 14357120 | 65.8% |

**Appendix 11.** Dispensed to written prescription ratio for ATC S drugs (sensory organs agents) by patient characteristics in Hungary in the period from 1 January 2012 to 30 September 2015

| Patient characteristics | | Written prescriptions | Dispensed prescriptions | Percentage of the dispensed prescriptions |
| --- | --- | --- | --- | --- |
| Age groups (years) | 18-44 | 344379 | 228643 | 66.4% |
|  | 45-64 | 536539 | 362283 | 67.5% |
|  | 65 and above | 745722 | 522697 | 70.1% |
| Sex | Male | 543743 | 372326 | 68.5% |
|  | Female | 1082897 | 741297 | 68.5% |
| Exemption certificate | Yes | 265041 | 198547 | 74.9% |
|  | No | 1361599 | 915076 | 67.2% |
| Total | | 1626640 | 1113623 | 68.5% |

**Appendix 12.** Dispensed to written prescription ratio for ATC V drugs (various agents) by patient characteristics in Hungary in the period from 1 January 2012 to 30 September 2015

| Patient characteristics | | Written prescriptions | Dispensed prescriptions | Percentage of the dispensed prescriptions |
| --- | --- | --- | --- | --- |
| Age groups (years) | 18-44 | 117297 | 71209 | 60.7% |
|  | 45-64 | 269348 | 164650 | 61.1% |
|  | 65 and above | 462899 | 280707 | 60.6% |
| Sex | Male | 382678 | 236836 | 61.9% |
|  | Female | 466866 | 279730 | 59.9% |
| Exemption certificate | Yes | 226234 | 150659 | 66.6% |
|  | No | 623310 | 365907 | 58.7% |
| Total | | 849544 | 516566 | 60.8% |

**Appendix 13.** Dispensed to written prescription ratio by ATC groups in Hungary in the period from 1 January 2012 to 30 September 2015

|  | Median | Limits of interquartile range | Interquartile range |
| --- | --- | --- | --- |
| Alimentary tract and metabolism | 1.09 | 0.88 - 1.22 | 0.34 |
| Blood and blood forming organs | 1.09 | 0.86 - 1.22 | 0.36 |
| Cardiovascular system | 1.09 | 0.84 - 1.26 | 0.42 |
| Dermatologicals | 1.08 | 0.88 - 1.25 | 0.37 |
| Genitourinary system and sex hormones | 1.07 | 0.83 - 1.24 | 0.41 |
| Systemic hormonal preparations* | 1.06 | 0.93 - 1.16 | 0.23 |
| Antiinfectives for systemic use | 1.04 | 0.96 - 1.1 | 0.14 |
| Musculoskeletal system | 1.07 | 0.89 - 1.18 | 0.29 |
| Nervous system | 1.07 | 0.87 - 1.19 | 0.32 |
| Respiratory system | 1.07 | 0.88 - 1.2 | 0.32 |
| Sensory organs | 1.05 | 0.84 - 1.22 | 0.38 |
| Various | 1.1 | 0.79 - 1.43 | 0.64 |
| Altogether^ | 1.08 | 0.87 - 1.22 | 0.35 |

** Excluding sex hormones and insulin*

*^ ATC groups of “Antineoplastic and immunomodulating agents” and “Antiparasitic products, insecticides, and repellents” were not studied*

**Appendix 14**. Deviations of the investigated SDWR parameters’ distribution from normal distribution according to Kolmogorov-Smirnov test by ATC groups.

|  | Non-transformed SDWR | | Box-Cox transformed SDWR | |
| --- | --- | --- | --- | --- |
|  | D | p-value | D | p-value |
| Alimentary tract and metabolism | 0.097543 | <0.001 | 0.001973 | 0.200 |
| Blood and blood forming organs | 0.099504 | <0.001 | 0.002017 | 0.200 |
| Cardiovascular system | 0.088931 | <0.001 | 0.002375 | 0.200 |
| Dermatologicals | 0.049958 | <0.001 | 0.004532 | 0.004 |
| Genitourinary system and sex hormones | 0.071367 | <0.001 | 0.006203 | <0.001 |
| Systemic hormonal preparations* | 0.106909 | <0.001 | 0.004013 | 0.019 |
| Antiinfectives for systemic use | 0.171842 | <0.001 | 0.001804 | 0.200 |
| Musculoskeletal system | 0.090163 | <0.001 | 0.001888 | 0.200 |
| Nervous system | 0.100263 | <0.001 | 0.002051 | 0.200 |
| Respiratory system | 0.07805 | <0.001 | 0.002256 | 0.200 |
| Sensory organs | 0.059144 | <0.001 | 0.008493 | <0.001 |
| Various | 0.037208 | <0.001 | 0.021532 | <0.001 |
| Altogether^ | 0.095995 | <0.001 | 0.002128 | 0.2 |

** Excluding sex hormones and insulin*

*^ ATC groups of “Antineoplastic and immunomodulating agents” and “Antiparasitic products, insecticides, and repellents” were not studied*

**Appendix 15.**

Distribution of general medical practice specific age-, gender-, and exemption certificate-standardized dispensed to written prescription ratios among Hungarian adults (not transformed) and their Box-Cox normalized (transformed) values in the period from 1 January 2012 to 30 September 2015 for ATC A (Alimentary tract and metabolism) group of medicine


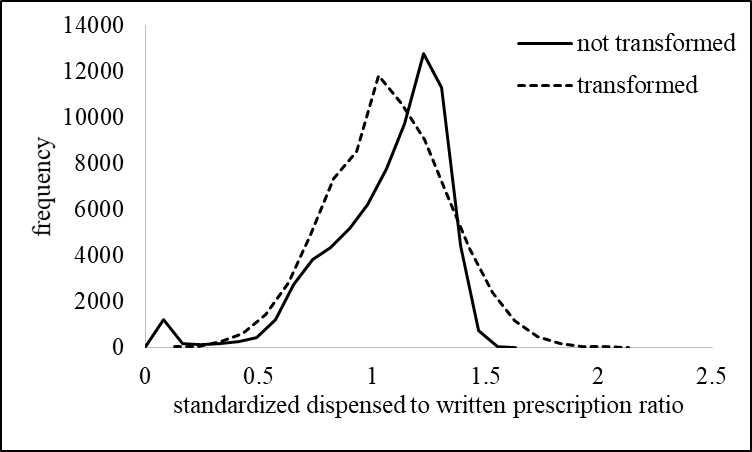


Distribution of general medical practice specific age-, gender-, and exemption certificate-standardized dispensed to written prescription ratios among Hungarian adults (not transformed) and their Box-Cox normalized (transformed) values in the period from 1 January 2012 to 30 September 2015 for ATC B (Blood and blood forming organs) group of medicine


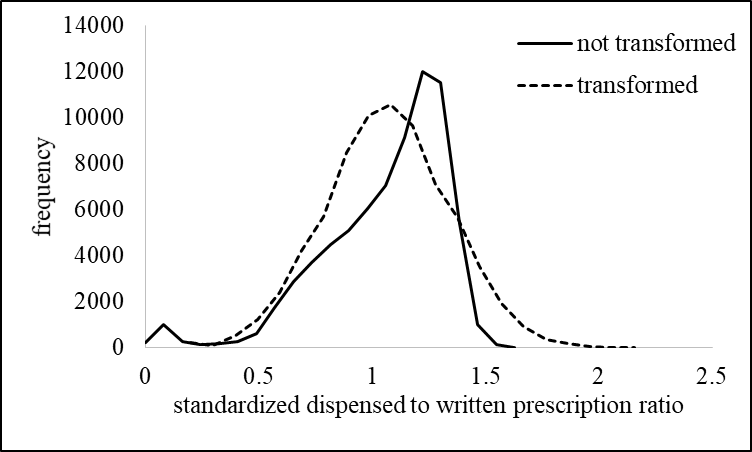


Distribution of general medical practice specific age-, gender-, and exemption certificate-standardized dispensed to written prescription ratios among Hungarian adults (not transformed) and their Box-Cox normalized (transformed) values in the period from 1 January 2012 to 30 September 2015 for ATC C (Cardiovascular system) group of medicine


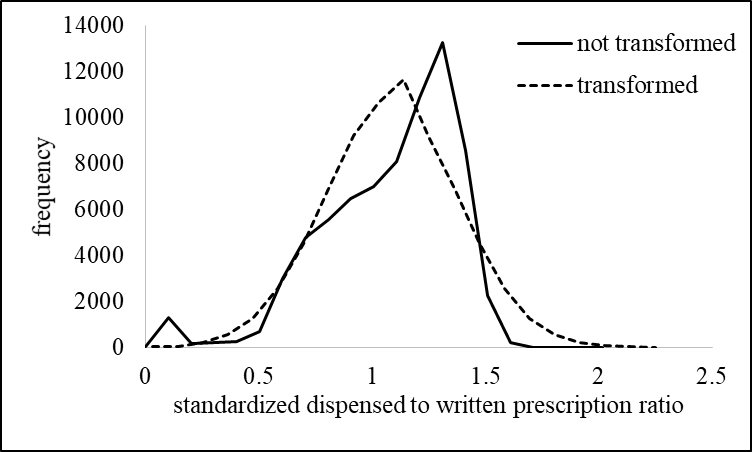


Distribution of general medical practice specific age-, gender-, and exemption certificate-standardized dispensed to written prescription ratios among Hungarian adults (not transformed) and their Box-Cox normalized (transformed) values in the period from 1 January 2012 to 30 September 2015 for ATC D (Dermatologicals) group of medicine


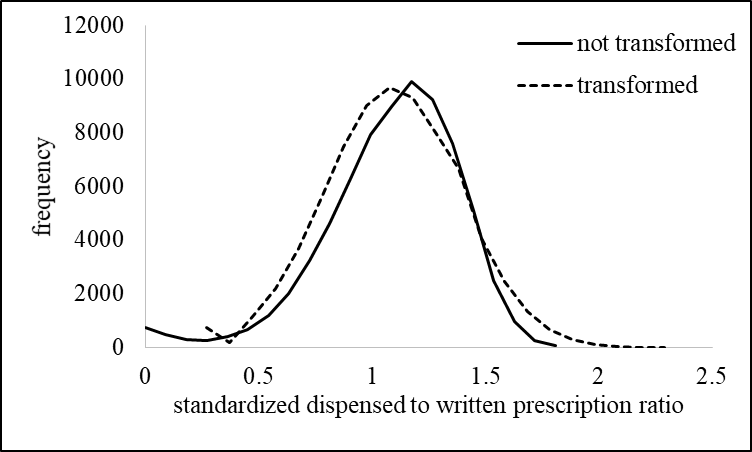


Distribution of general medical practice specific age-, gender-, and exemption certificate-standardized dispensed to written prescription ratios among Hungarian adults (not transformed) and their Box-Cox normalized (transformed) values in the period from 1 January 2012 to 30 September 2015 for ATC G (Genitourinary system and sex hormones) group of medicine


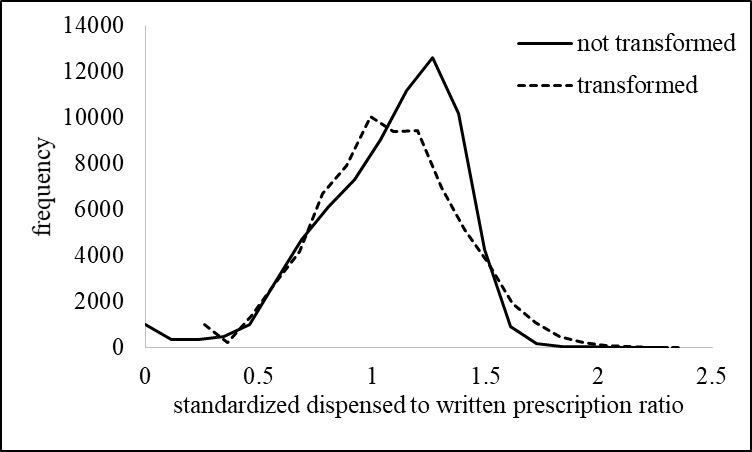


Distribution of general medical practice specific age-, gender-, and exemption certificate-standardized dispensed to written prescription ratios among Hungarian adults (not transformed) and their Box-Cox normalized (transformed) values in the period from 1 January 2012 to 30 September 2015 for ATC H (Systemic hormonal preparations*) group of medicine


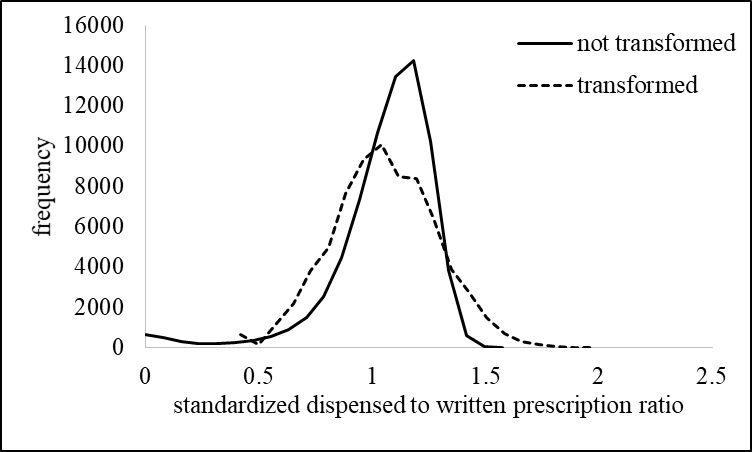


** Excluding sex hormones and insulin*

Distribution of general medical practice specific age-, gender-, and exemption certificate-standardized dispensed to written prescription ratios among Hungarian adults (not transformed) and their Box-Cox normalized (transformed) values in the period from 1 January 2012 to 30 September 2015 for ATC J (Antiinfectives for systemic use) group of medicine


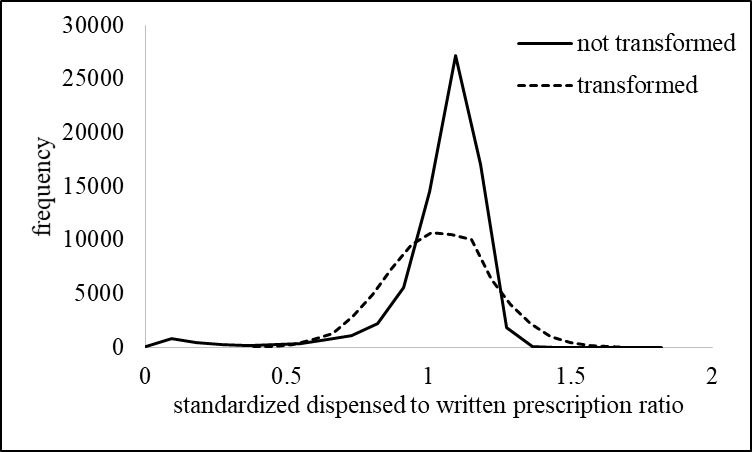


Distribution of general medical practice specific age-, gender-, and exemption certificate-standardized dispensed to written prescription ratios among Hungarian adults (not transformed) and their Box-Cox normalized (transformed) values in the period from 1 January 2012 to 30 September 2015 for ATC M (Musculoskeletal system) group of medicine


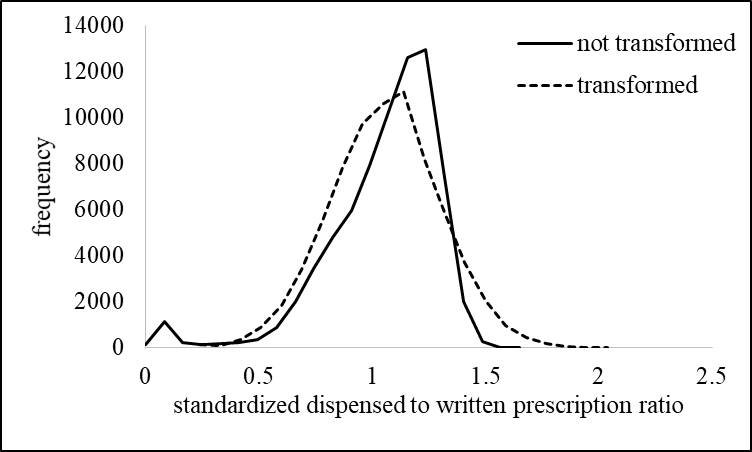


Distribution of general medical practice specific age-, gender-, and exemption certificate-standardized dispensed to written prescription ratios among Hungarian adults (not transformed) and their Box-Cox normalized (transformed) values in the period from 1 January 2012 to 30 September 2015 for ATC N (Nervous system) group of medicine


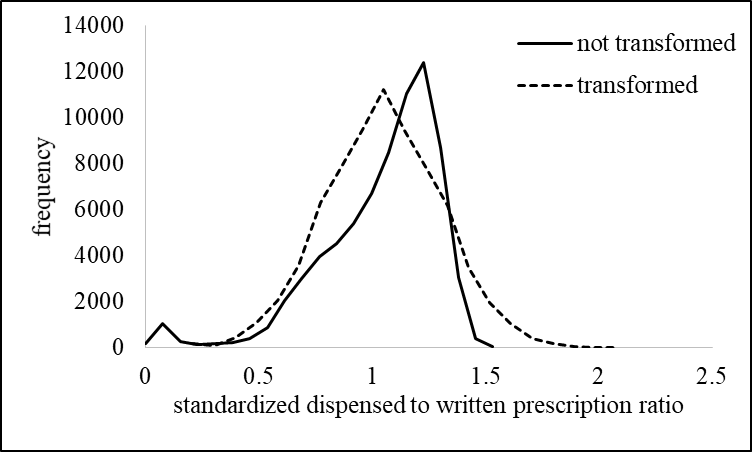


Distribution of general medical practice specific age-, gender-, and exemption certificate-standardized dispensed to written prescription ratios among Hungarian adults (not transformed) and their Box-Cox normalized (transformed) values in the period from 1 January 2012 to 30 September 2015 for ATC R (Respiratory system) group of medicine


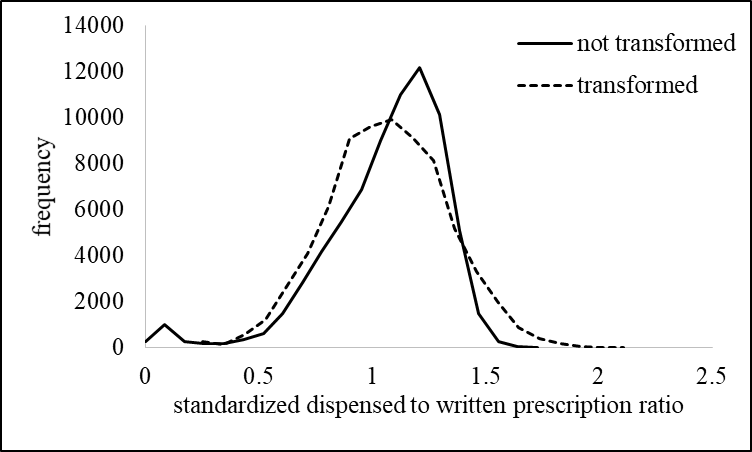


Distribution of general medical practice specific age-, gender-, and exemption certificate-standardized dispensed to written prescription ratios among Hungarian adults (not transformed) and their Box-Cox normalized (transformed) values in the period from 1 January 2012 to 30 September 2015 for ATC S (Sensory organs) group of medicine


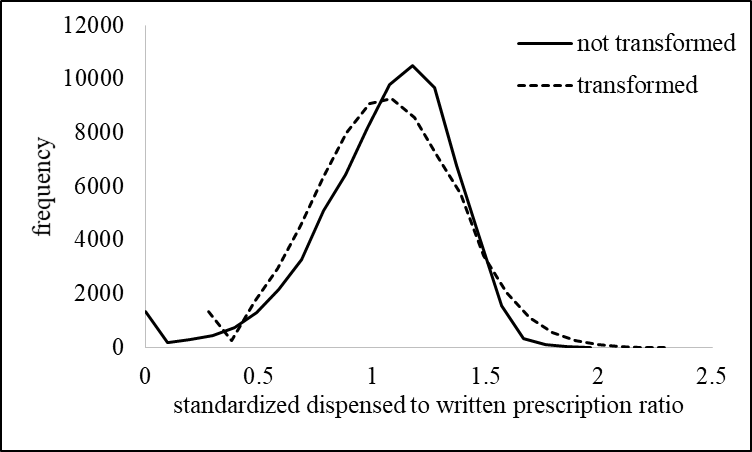


Distribution of general medical practice specific age-, gender-, and exemption certificate-standardized dispensed to written prescription ratios among Hungarian adults (not transformed) and their Box-Cox normalized (transformed) values in the period from 1 January 2012 to 30 September 2015 for ATC V (Various) group of medicine


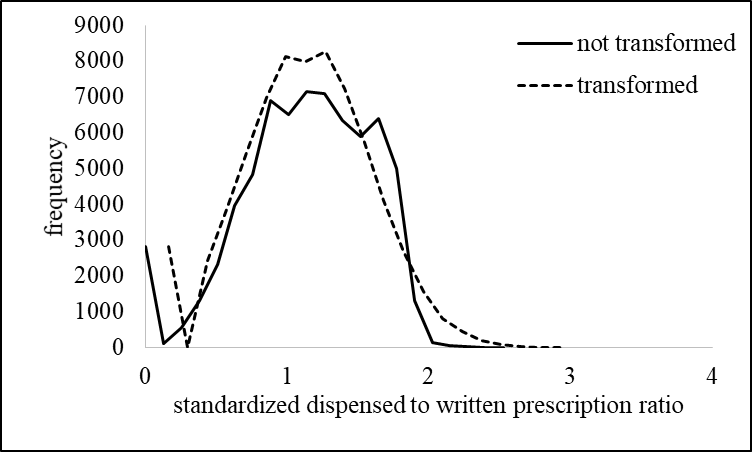


Distribution of general medical practice specific age-, gender-, and exemption certificate-standardized dispensed to written prescription ratios among Hungarian adults (not transformed) and their Box-Cox normalized (transformed) values in the period from 1 January 2012 to 30 September 2015 for each ATC group of medicine without “Antineoplastic and immunomodulating agents” and “Antiparasitic products, insecticides, and repellents”


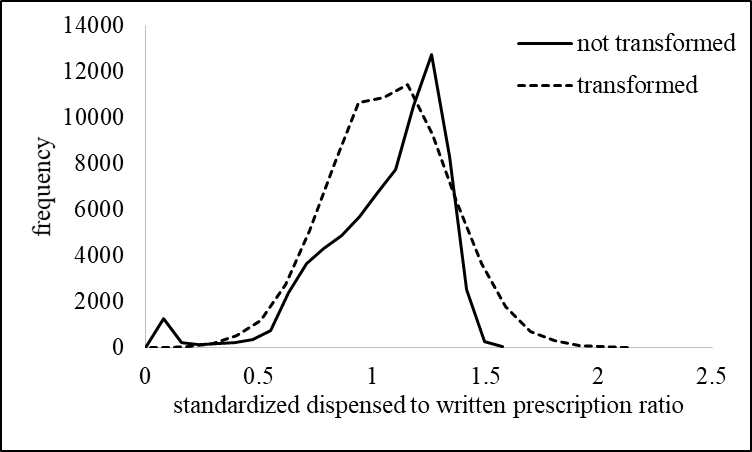


**Appendix 16.** Associations between general medical practice (GMP) characteristics and normalized* age-, gender, and exemption certificate-standardized dispensed to written prescription ratio among Hungarian adults by ATC groups according to generalized linear regression analysis controlled for time in the period from 1 January 2012 to 30 September 2015 (linear regression coefficient; lower and upper limit of the 95% confidence interval for standardized linear regression coefficient)

|  | ATC A | ATC B | ATC C | ATC D | ATC G | ATC H | ATC J | ATC M | ATC N | ATC R | ATC S | ATC V |
| --- | --- | --- | --- | --- | --- | --- | --- | --- | --- | --- | --- | --- |
| Relative education (standardized continuous parameter) | -0.442  -0.469  -0.415 | -0.470  -0.498  -0.442 | -0.487  -0.518  -0.457 | -0.216  -0.247  -0.184 | -0.493  -0.525  -0.461 | -0.309  -0.334  -0.285 | -0.091  -0.111  -0.072 | -0.320  -0.346  -0.295 | -0.456  -0.482  -0.430 | -0.446  -0.473  -0.419 | -0.299  -0.332  -0.266 | -0.266  -0.316  -0.216 |
| Vacant GP position / filled GP position | -0.186-0.197  -.0.176 | -0.184  -0.195  -0.172 | -0.211  -0.223  -0.199 | -0.190  -0.202  -0.177 | -0.182  -0.195  -0.170 | -0.134  -0.144  -0.125 | -0.128  -0.136  -0.120 | -0.178  -0.188  -0.168 | -0.179  -0.189  -0.168 | -0.185  -0.196  -0.174 | -0.196  -0.209  -0.182 | -0.171  -0.192  -0.151 |
| Urban / rural | -.099  -0.104  -0.095 | -0.090  -0.094  -0.085 | -0.100  -0.105  -0.095 | -0.078  -0.084  -0.073 | -0.081  -0.086  -0.075 | -0.050  -0.054  -0.046 | -0.030  -0.033  -0.026 | -0.093  -0.097  -0.088 | -0.099  -0.104  -0.095 | -0.096  -0.101  -0.092 | -0.075  -0.081  -0.069 | -0.050  -0.059  -0.042 |
| X-800 GMP size / 1601-2000 GMP size | 0.051  0.040  0.062 | 0.027  0.016  0.038 | 0.058  0.046  0.070 | 0.046  0.033  0.059 | 0.031  0.018  0.045 | 0.021  0.011  0.031 | -0.016  -0.024  -0.008 | 0.049  0.039  0.059 | 0.045  0.034  0.055 | 0.045  0.34  0.056 | 0.027  0.013  0.041 | 0.008  -0.014  0.030 |
| 801-1200 GMP size / 1601-2000 GMP size | 0.028  0.022  0.034 | 0.017  0.011  0.023 | 0.034  0.028  0.041 | 0.022  0.015  0.028 | 0.030  0.023  0.037 | 0.026  0.021  0.032 | -0.012  -0.017  -0.008 | 0.025  0.020  0.031 | 0.029  0.023  0.035 | 0.029  0.023  0.035 | 0.015  0.008  0.022 | 0.016  0.005  0.027 |
| 1201-1600 GMP size / 1601-2000 GMP size | 0.016  0.012  0.021 | 0.011  0.006  0.015 | 0.021  0.016  0.026 | 0.015  0.009  0.020 | 0.016  0.011  0.021 | 0.015  0.011  0.019 | -0.006  -0.009  -0.002 | 0.012  0.008  0.016 | 0.014  0.010  0.018 | 0.014  0.010  0.019 | 0.013  0.008  0.019 | 0.022  0.014  0.030 |
| 2001-X GMP size / 1601-2000 GMP size | -0.016  -0.021  -0.011 | -0.013  -0.018  -0.008 | -0.013  -0.018  -0.007 | -0.027  -0.033  -0.021 | -0.022  -0.028  -0.016 | -0.015  -0.020  -0.011 | -0.004  -0.007  0.000 | -0.013  -0.018  -0.008 | -0.014  -0.019  -0.009 | -0.017  -0.023  -0.012 | -0.019  -0.025  -0.012 | -0.013  -0.022  -0.004 |
| Baranya county / Budapest | 0.080  0.070  0.091 | 0.108  0.097  0.119 | 0.089  0.078  0.101 | 0.024  0.012  0.037 | 0.074  0.062  0.086 | 0.014  0.005  0.023 | 0.021  0.013  0.029 | 0.077  0.067  0.086 | 0.079  0.069  0.089 | 0.023  0.013  0.033 | -0.008  -0.021  0.005 | -0.020  -0.039  -0.002 |
| Bács-Kiskun county / Budapest | 0.059  0.049  0.069 | 0.066  0.056  0.076 | 0.044  0.032  0.055 | 0.078  0.066  0.090 | 0.047  0.035  0.059 | 0.066  0.057  0.075 | 0.089  0.082  0.097 | 0.073  0.063  0.086 | 0.057  0.047  0.067 | 0.055  0.045  0.065 | 0.053  0.041  0.065 | 0.012  -0.006  0.031 |
| Békés county / Budapest | 0.052  0.041  0.063 | 0.054  0.042  0.065 | -0.000  -0.013  0.012 | 0.083  0.071  0.096 | 0.049  0.036  0.061 | 0.052  0.042  0.061 | 0.053  0.045  0.061 | 0.049  0.039  0.060 | 0.043  0.033  0.054 | 0.011  0.001  0.022 | 0.040  0.027  0.053 | 0.083  0.063  0.103 |
| Borsod-Abaúj-Zemplén county / Budapest | 0.010  0.001  0.018 | 0.035  0.026  0.044 | -0.031  -0.040  -0.021 | -0.054  -0.064  -0.044 | 0.031  0.020  0.041 | -0.017  -0.025  -0.009 | -0.029  -0.036  -0.023 | 0.004  -0.004  0.012 | 0.003  -0.006  0.011 | -0.027  -0.035  -0.018 | -0.018  -0.029  -0.007 | -0.040  -0.056  -0.024 |
| Csongrád county / Budapest | 0.029  0.019  0.039 | 0.030  0.020  0.041 | 0.010  -0.001  0.022 | -0.031  -0.043  -0.019 | 0.013  0.000  0.025 | 0.037  0.027  0.046 | 0.049  0.042  0.057 | 0.038  0.028  0.047 | 0.026  0.016  0.036 | 0.021  0.011  0.031 | 0.027  0.014  0.039 | 0.043  0.024  0.061 |
| Fejér county / Budapest | -0.128-0.139  -0.118 | -0.119  -0.130  -0.108 | -0.150  -0.162  -0.138 | -0.091  -0.104  -0.079 | -0.114  -0.126  -0.101 | -0.088  -0.097  -0.079 | -0.066  -0.074  -0.058 | -0.103  -0.113  -0.093 | -0.112  -0.122  -0.102 | -0.131  -0.142  -0.121 | -0.091  -0.104  -0.078 | -0.127  -0.146  -0.107 |
| Győr-Moson-Sopron county / Budapest | 0.014  0.004  0.024 | 0.022  0.0110.032 | -0.030  -0.041  -0.018 | 0.012  0.000  0.024 | 0.005  -0.007  0.018 | 0.020  0.011  0.029 | 0.062  0.055  0.070 | 0.040  0.030  0.049 | 0.018  0.008  0.028 | 0.000  -0.011  0.010 | 0.006  -0.007  0.018 | 0.048  0.030  0.067 |
| Hajdú-Bihar county / Budapest | -0.012  -0.021  -0.002 | -0.002  -0.012  0.008 | -0.061  -0.072  -0.050 | -0.018  -0.030  -0.007 | 0.020  0.009  0.032 | 0.012  0.004  0.021 | -0.013  -0.021  -0.006 | -0.004  -0.013  0.006 | -0.004  -0.013  0.006 | -0.031  -0.041  -0.021 | -0.022  -0.034,-0.010 | 0.008  -0.009  0.026 |
| Heves county / Budapest | -0.006  -0.017  0.006 | 0.003  -0.009  0.015 | -0.045  -0.058  -0.033 | -0.038  -0.051  -0.024 | -0.007  -0.021  0.006 | -0.032  -0.042  -0.021 | -0.056  -0.065  -0.048 | -0.036  -0.047  -0.025 | -0.016  -0.027  -0.005 | -0.030  -0.041  -0.019 | -0.052  -0.066  -0.038 | -0.031  -0.051  -0.010 |
| Jász-Nagykun-Szolnok county / Budapest | 0.059  0.048  0.070 | 0.069  0.057  0.080 | 0.042  0.030  0.054 | 0.010  -0.003  0.023 | 0.083  0.071  0.096 | 0.077  0.067  0.086 | -0.005  -0.013  0.003 | 0.028  0.018  0.039 | 0.054  0.043  0.064 | 0.023  0.012  0.034 | 0.017  0.003  0.030 | 0.024  0.004  0.043 |
| Komárom-Esztergom county / Budapest | 0.085  0.073  0.097 | 0.102  0.090  0.114 | 0.066  0.052  0.079 | 0.035  0.021  0.049 | 0.116  0.102  0.130 | 0.076  0.066  0.087 | 0.019  0.011  0.028 | 0.079  0.068  0.090 | 0.064  0.053  0.076 | 0.077  0.065  0.089 | 0.028  0.014  0.043 | 0.109  0.087  0.131 |
| Nógrád county / Budapest | -0.012  -0.025  0.001 | -0.006  -0.020  0.008 | -0.021  -0.036  -0.006 | -0.074  -0.089  -0.058 | -0.008  -0.024  0.008 | -0.028  -0.039  -0.016 | -0.044  -0.053  -0.034 | -0.035  -0.047  -0.022 | -0.031  -0.044  -0.018 | -0.069  -0.082  -0.056 | -0.031  -0.047  -0.014 | 0.035  0.010  0.060 |
| Pest county / Budapest | 0.004  -0.004  0.012 | 0.006  -0.002  0.014 | -0.011  -0.020  -0.003 | -0.007  -0.017  0.002 | 0.008  -0.001  0.018 | -0.005  -0.012  0.002 | 0.015  0.009  0.021 | 0.007  0.000  0.015 | 0.003  -0.005  0.010 | 0.002  -0.005  0.010 | 0.005  -0.005  0.015 | 0.046  0.031  0.060 |
| Somogy county / Budapest | 0.095  0.083  0.106 | 0.107  0.096  0.119 | 0.058  0.045  0.071 | 0.139  0.126  0.152 | 0.063  0.050  0.077 | 0.073  0.063  0.083 | 0.115  0.107  0.123 | 0.102  0.091  0.112 | 0.076  0.066  0.087 | 0.087  0.075  0.098 | 0.098  0.084  0.111 | 0.082  0.061  0.103 |
| Szabolcs-Szatmár-Bereg county / Budapest | 0.094  0.084  0.104 | 0.123  0.113  0.133 | 0.075  0.064  0.087 | 0.017  0.005  0.029 | 0.107  0.095  0.119 | 0.055  0.046  0.064 | 0.032  0.025  0.040 | 0.075  0.066  0.085 | 0.081  0.072  0.091 | 0.049  0.039  0.059 | 0.021  0.008  0.033 | 0.083  0.065  0.101 |
| Tolna county / Budapest | 0.068  0.055  0.081 | 0.112  0.099  0.125 | 0.044  0.030  0.059 | 0.066  0.051  0.081 | 0.086  0.071  0.101 | 0.041  0.030  0.053 | 0.090  0.081  0.099 | 0.090  0.078  0.102 | 0.053  0.041  0.066 | 0.037  0.024  0.050 | 0.064  0.048  0.080 | -0.057  -0.080  -0.034 |
| Vas county / Budapest | 0.102  0.90  0.114 | 0.114  0.102  0.127 | 0.104  0.090  0.117 | 0.034  0.020  0.048 | 0.065  0.051  0.079 | 0.004  -0.007  0.015 | 0.035  0.026  0.044 | 0.101  0.090  0.112 | 0.084  0.073  0.096 | 0.084  0.072  0.096 | -0.010  -0.025  -0.005 | 0.057  0.034  0.079 |
| Veszprém county / Budapest | 0.010  -0.001  0.022 | 0.009  -0.003  0.020 | -0.014  -0.026  -0.001 | 0.092  0.079  0.105 | -0.002  -0.015  0.012 | 0.020  0.010  0.030 | 0.049  0.041  0.058 | 0.039  0.029  0.050 | 0.016  0.005  0.027 | 0.016  0.005  0.027 | 0.022  0.008  0.035 | 0.072  0.051  0.093 |
| Zala county / Budapest | 0.050  0.038  0.062 | 0.038  0.026  0.051 | -0.019  -0.032  -0.005 | 0.093  0.079  0.107 | 0.029  0.015  0.043 | 0.025  0.014  0.035 | 0.057  0.048  0.066 | 0.060  0.048  0.071 | 0.048  0.037  0.060 | 0.037  0.025  0.049 | 0.060  0.046  0.075 | -0.005  -0.027  0.017 |
| P-value for goodness of fit (Pearson Chi- square) | 0.059 | 0.063 | 0.074 | 0.081 | 0.083 | 0.047 | 0.032 | 0.051 | 0.055 | 0.058 | 0.088 | 0.188 |

** Box-Cox transformation*
